# The dual mediating effect of self-efficacy and motivation between conscientiousness and academic performance of university students in China: the moderated mediation effect of gender

**DOI:** 10.3389/fpsyg.2025.1538200

**Published:** 2025-04-28

**Authors:** Jianan Li, Tingting Ma, Chang Seek Lee

**Affiliations:** ^1^School of Digital Economics and Management, Software Engineering Institute of Guangzhou, Guangzhou, China; ^2^Department of Lifelong Education, Hanseo University, Seosan, Republic of Korea

**Keywords:** academic motivation, academic performance, academic self-efficacy, conscientiousness, gender, moderated mediation effect

## Abstract

In recent decades, there has been extensive research on the impact of personality on academic achievement, among which conscientiousness was verified to be a stable contributor. This study aims to clarify the moderated mediation relationship among conscientiousness, gender, academic self-efficacy, academic motivation, and academic performance. To achieve this purpose, the current study proposed a hypothesized model and verified it through a random sampling survey. With the informed consent of the respondents, the survey was conducted on 304 Chinese university students through the Wenjuanxing platform. For data research and analysis tools, SPSS and PROCESS macro were used, specifically, descriptive statistics analysis, reliability analysis, correlation analysis, and SPSS PROCESS macro model 83. The results indicated that conscientiousness, academic self-efficacy, academic motivation, and academic performance were positively correlated. Furthermore, gender moderated the mediating relationship from conscientiousness to academic self-efficacy, academic motivation, and academic performance. Such research results provided new ideas on how to improve the academic performance of college students, namely, focusing on and improving their academic self-efficacy and academic motivation. Meanwhile, it also provides more targeted help for the gender-differentiated characteristics of college students.

## 1 Introduction

In the field of educational psychology, the exploration of predictors of academic performance (AP) has an ongoing history (Lavin, 1965). More recently, as research on the Big Five theory of personality has deepened, conscientiousness was repeatedly verified to be a solid contributor to AP (Trautwein et al., 2009). Meanwhile, other concepts of cognitive psychology, such as academic self-efficacy (ASE) and academic motivation (AM), also facilitated the further explanation of AP. However, insufficient research was able to elucidate the causal relationships among conscientiousness, ASE, AM, and AP within the framework of moderated mediation models. The lack of clarity about the causal relationships among these important variables will further hinder the development and application of effective educational interventions. In this case, it is urgent to clarify the relationships among these variables, and the purpose of this study is to explore the moderated mediation effect between AP and its main contributors.

Self-efficacy (SE), defined as individuals’ judgments of one’s capabilities to organize and execute courses of action required to attain designated types of performances (Bandura, 1986a), was frequently used in the field of educational psychology, which was embodied as ASE. ASE was announced to be correlated with conscientiousness (Lee and Klein, 2002) and AP (Di Giunta et al., 2013). Besides, another important psychological concept, academic motivation, which was composed of intrinsic motivation and extrinsic motivation (Deci and Ryan, 2013), also presented the same characteristics as SE’s connection with conscientiousness and AP. It was verified that more conscientious students were more intrinsically motivated (McGeown et al., 2014) and consequently enjoyed a higher level of academic achievement (Kırkağaç and Öz, 2017). Besides, there was also a strong causal relationship between ASE and AM (McGeown et al., 2014). Based on the above research results, it is very likely that ASE and AM separately and together play mediating roles in the causal relationship between conscientiousness and academic achievement.

Additionally, plenty of research results confirmed that gender differences had a significant impact on people’s level of SE; specifically, male students had a stronger sense of efficacy in learning science, while female students had more powerful efficacy in learning liberal arts (Huang, 2013). Another study also discovered that the effects of prior experiences appeared to differentiate men and women in the effects of occupational efficacy (Scott and Ciani, 2008). In this sense, gender is possible to moderate the dual mediation effect among conscientiousness, ASE, AM, and AP. However, to date, no research results have ever demonstrated such results, and therefore, this study aims to fill this gap. To verify the intrinsic relationship between the above core variables, research questions were set as the following. First, what are the interrelationships among conscientiousness, ASE, AM, and AP? Second, is there a moderated mediation effect of gender in the dual mediation effect of ASE and AM between conscientiousness and AP?

## 2 Literature review

### 2.1 Conscientiousness and academic performance

Rooted in the Big Five theory of personality, conscientiousness was defined as being orderly, dependable, and diligent (Costa and McCrae, 2008). Indeed, conscientiousness represented one’s traits, which included competence, order, dutifulness, achievement striving, self-discipline, and deliberation (McGhee et al., 2007), and these qualities were consistent with the academic orientation.

As traditionally used, the term “academic performance” refers to some method of expressing a student’s scholastic standing (Lavin, 1965). In higher education, students’ performance was usually expressed in terms of grade point average (GPA), which was the key criterion for postgraduate selection and graduate employment (Strenze, 2007).

Academic research on the positive correlation between conscientiousness and AP was extensive and sufficient (Vedel, 2014). The fact that conscientious students could achieve greater academic success had been verified repeatedly, both in high school (Trautwein et al., 2009) and at college levels (Chamorro-Premuzic and Furnham, 2008). Besides, compared with other personality of the Big Five theory, such as extraversion, agreeableness, neuroticism, and openness, conscientiousness was the strongest and most reliable predictor of academic achievement (Trapmann et al., 2007). However, the academic community had not yet reached any consensus on the mediating mechanism between conscientiousness and AP. Therefore, what mediates the relationship between these two variables needs further exploration.

### 2.2 Dual mediating effect of academic self-efficacy and academic motivation

First, according to the Social Cognitive Theory, self-efficacy was represented as a situational heterogeneous concept affecting individuals’ construction of activities, goals, and persistent choices (Bandura, 1986a). In the educational context, academic self-efficacy refers to the judgment of a learner’s ability to achieve the desired performance in the academic field (Bandura, 1997).

Furthermore, research results in recent decades have confirmed that ASE was positively correlated with both conscientiousness (Chen et al., 2001) and AP (Zeinalipour, 2022). More importantly, it was verified that more conscientious students contributed to a higher level of ASE (Lee and Klein, 2002), and students with a stronger sense of ASE would positively influence students’ academic achievement at last (Di Giunta et al., 2013). In other words, academic self-efficacy was of obvious causal relationships with conscientiousness and AP, and this connection laid the foundation for ASE to become a potential mediating variable to bridge the above path.

Second, academic motivation, which originated from the Self-Determination Theory, was referred to as the different loci of causality with regard to engagement in learning, which was often differentiated from extrinsic to intrinsic motivation (Ryan and Deci, 2000). More specifically, academic intrinsic motivation often refers to engaging in academic activities for their own sake, while extrinsic motivation means engaging in learning due to external factors (McGeown et al., 2014).

More importantly, AM was also closely linked to both conscientiousness (Apostolov and Geldenhuys, 2022) and AP (Gnambs and Hanfstingl, 2016). Furthermore, recent studies have testified that personality traits predicted 20.6% of the variance in intrinsic motivation and 5.1% in extrinsic motivation; specifically, only conscientiousness significantly predicted both intrinsic and extrinsic motivation (McGeown et al., 2014). Besides, AM accounted for 10% of the variance in academic achievement in university students based on a Turkish study (Kırkağaç and Öz, 2017). The strong causal relationship between AM, conscientiousness, and AP has been widely confirmed by academics. Consequently, AM would potentially play a mediating role in the connection between conscientiousness and AP.

Finally, ASE also correlated with motivation (Bandura, 1986b) and had repeatedly been verified to be a significant predictor of both intrinsic and extrinsic motivation (McGeown et al., 2014), which meant that the path from ASE to AM is also viable. Therefore, in together, based on the above findings on the causal relationships among conscientiousness, ASE, AM, and AP, the path starting from conscientiousness to AP through ASE and AM seems logical and feasible. Previous studies had attempted to incorporate conscientiousness, ASE, and AP with mediation models for research (Cupani and Pautassi, 2013), but there was still limited research on the assumed dual mediation in this article.

### 2.3 Moderating effect of gender

In the field of educational research, demographic information, especially gender, had been frequently used as a moderating variable (Choma et al., 2010) and was of a solid research foundation. Moreover, the research on gender serving as a moderator in its relationship with self-efficacy showed diversity, and the results of gender moderating ASE were not uniform. Some studies found no difference between male and female students, while others suggested that male students may have higher levels of self-efficacy and better science learning outcomes (Nurhasnah et al., 2022). For instance, a meta-analysis of 187 studies (including 247 individual studies, N = 68,429) on gender differences in ASE found that women reported a higher level of self-efficacy in the arts than men; meanwhile, men reported stronger self-efficacy in the sciences than women (Huang, 2013). In addition, studies tested how gender might influence the relationship between self-efficacy, academic success, and science identity (Miles and Naumann, 2021).

Overall, these findings highlighted the significance of considering gender when examining ASE, as it played an important role in shaping students’ educational beliefs and motivations. Based on previous research, it seems plausible that gender may play a moderated mediation role in the pathway from conscientiousness to ASE, AM, and AP.

## 3 Research methodology

### 3.1 Research model

**Figure 1:**
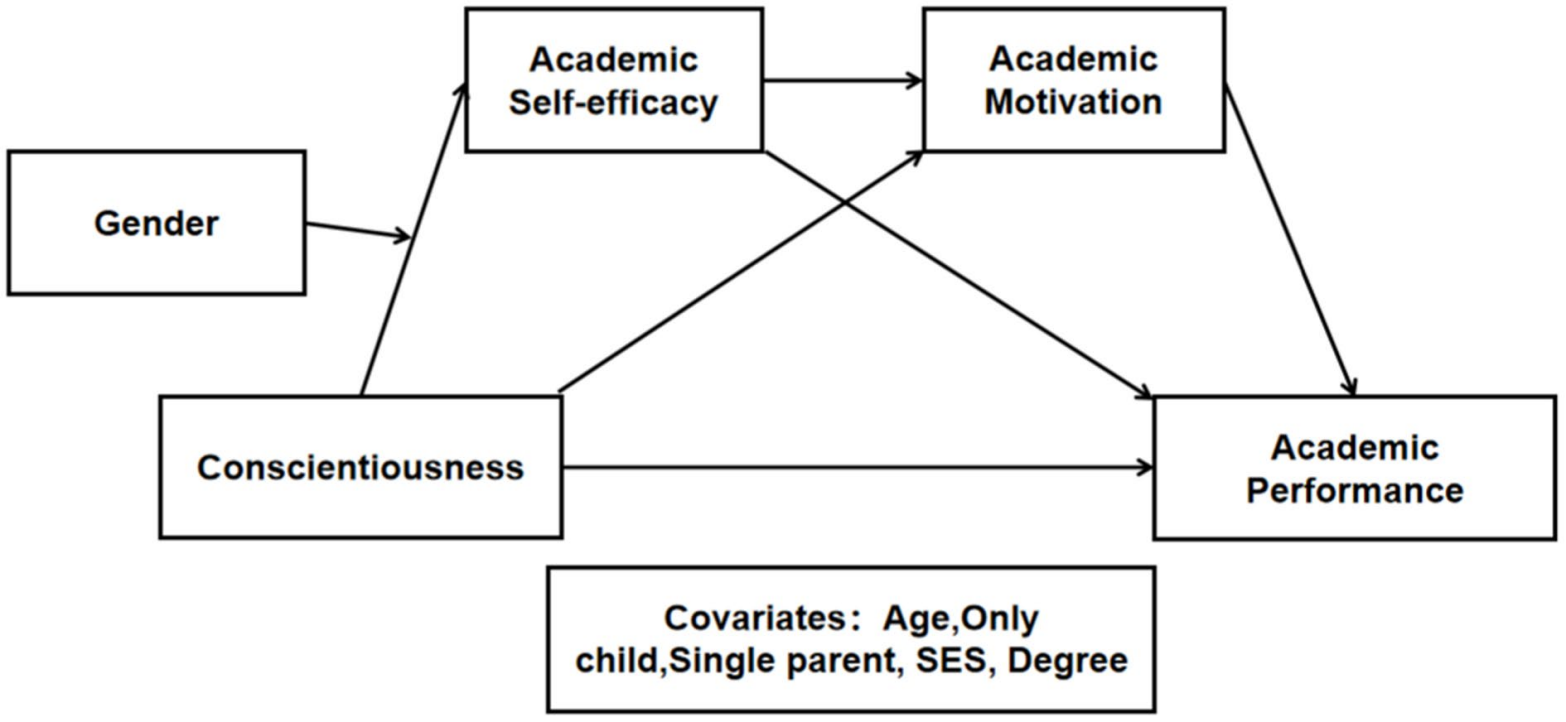
Proposed research model.

The hypothesized model (Figure 1) proposed in this study was used to verify the dual mediating role of ASE and AM in the path from conscientiousness to AP and, further, to prove the moderated dual mediating role of gender in the above relationship.

### 3.2 Respondents of the study

The respondents of this study were 304 college students from China, and the sample covered 32 of the 34 provincial-level administrative regions (provinces, autonomous regions, municipalities directly under the central government, and special administrative regions) in China. The overall characteristics of the respondents were as follows: by gender, 40.1% were male and 59.9% were female and by age, 12.5% of the respondents were no older than 19 years of age, 30.6% were 20 years of age, 26.6% were 21 years of age, 22.7% were 22 years of age, and 7.6% were older than 22 years. From the perspective of only children, 41.4% of the respondents were not the only child, and 58.6% were the only child. In addition, 8.2% of the respondents came from single-parent families, and 91.8% came from two-parent families. In terms of socioeconomic status, 25.7% of the respondents came from wealthy families, 53.6% from average families, and 20.7% from less wealthy families. Regarding the participants’ current educational background, 49.7% were 4-year undergraduate students and 50.3% were 3-year college students.

### 3.3 Data gathering procedures

The sampling process was conducted from 17 June to 28 June 2024. The sampling subjects were 870,000 Chinese college students registered on the Wenjuanxing platform. The sampling method was simple random sampling. First, the computer software randomly selected 3,782 registered college students on the Wenjuanxing registration platform. Then, an invitation letter, including a consent letter and a questionnaire, was sent to invite these selected students. In the invitation letter, the purpose of the study, the research procedures, benefits, risks, confidentiality, voluntary participation, and the withdrawal terms were explained to potential participants. Only students who agreed to participate and confirmed the consent form were included in the formal sampling procedure. Afterwards, 1,323 students agreed to participate in the study who had confirmed the consent form. Then, the participants were asked to complete an online questionnaire through the Wenjuanxing platform within 20 minutes, and 1,040 participants submitted the results. Participants who answered fully and submitted the questionnaire will receive a 5-yuan reward. Next, the research team checked the results and deleted invalid questionnaires. Specifically, a total of 737 results were judged to be invalid. Finally, 304 participants were marked as valid results and used for further analysis.

### 3.4 Research instrument

#### 3.4.1 Conscientiousness

Originating from Costa and McCrae’s NEO-PI-R (Costa Jr and McCrae, 2000), the brief version of the Chinese Big Five Personality Inventory (CBF-PI-B) developed by Wang et al. (2011) was used to test conscientiousness. This scale has been proven to have good validity, including the sub-scale for conscientiousness, especially for Chinese students (Wang et al., 2010). This sub-scale of conscientiousness consisted of eight items. It was a 6-point Likert scale ranging from 1 point of “Not at all consistent” to 6 points of “Completely consistent.” Lower points indicated a weaker level of conscientiousness. Question 7 is rhetorical. There was a question like “Others think I am a cautious person.” The reliability of conscientiousness was high (Cronbach’s *α* = 0.819).

#### 3.4.2 Academic self-efficacy

To measure ASE, the scale originally developed by Midgley (2000) and modified by Chen et al. (2016) for the Chinese situation was used, which was of good validity (root mean square error of approximation (RMSEA) = 0.126, comparative fit index (CFI) = 0.958, tucker-lewis index (TLI) = 0.930, standardized root mean square residual (SRMR) = 0.031). This scale has a total of five items. It was a 5-point Likert scale ranging from 1 point of “strongly disagree” to 5 points of “strongly agree.” The higher the score, the higher the level of ASE. There was a question like “I’m certain I can master the skills taught in class this year.” The reliability of ASE was high (Cronbach’s α = 0.794).

#### 3.4.3 Academic motivation

To measure AM, the Short Academic Motivation Scale (SAMS) (Kotera et al., 2023) in Chinese (Song, 2014) was used. This scale was verified to have good validity (CFI = 0.92, TLI = 0.90, RMSEA = 0.08) and had a total of 14 items (Kotera et al., 2023). The scale included seven subscales, namely, intrinsic motivation to know, intrinsic motivation to accomplishments, intrinsic motivation to experience stimulation, identified regulation, introjected regulation, external regulation, and amotivation. There were questions like “Because college allows me to experience personal satisfaction in my quest for excellence in my studies.” It was a 7-point Likert scale ranging from 1 point of “does not correspond at all” to 7 points of “correspond exactly.” The SAMS instrument’s overall score is termed a Self Determination Index (SDI), which was a formula for calculating fractions: 2*(know+acc + stim)/3 + iden-((intro+reg)/2 + 2amo) (Zeng and Yao, 2023). The value of SDI ranged from −18 to +18. The higher the score, the more intrinsically motivated the participant tended to be. The reliability of AM was acceptable (Cronbach’s *α* = 0.703).

#### 3.4.4 Academic performance

To measure the AP of Chinese college students, the scale developed by Li and Lee (2023) was used. This scale was verified to have good validity (CFI = 0.99, TLI = 0.99, RMSEA = 0.07) and had a total of four items. It is a 5-point Likert scale ranging from 1 point of “strongly disagree” to 5 points of “strongly agree.” The higher the score, the higher the level of AP. There were questions like “My school professional curriculum has excellent grades.” This study’s reliability of AP was acceptable (Cronbach’s α = 0.738).

#### 3.4.5 Gender and covariates

For gender, participants indicated whether they were male (coded as 0) or female (1). Other personal characteristics affecting the mediating and dependent variables, such as age, only child, single parent, SES, and educational background, were set up as the control variables during the analysis process.

### 3.5 Statistical tools (for data analysis)

As for data analysis, the analysis tools were SPSS Ver.25 and SPSS PROCESS macro-Ver. 4.2. More specifically, frequency analysis was adopted to illustrate the characteristics of the participants. Next, the reverse question was addressed, and Cronbach’s α was tested to ensure the reliability of the variables. Afterward, to find out the answer to the first research question, Pearson’s bivariate correlation method was applied to demonstrate the relationships among the main variables. Finally, to address the second research question, the SPSS PROCESS macro model No.83 was undertaken to show the moderated dual mediation path among the variables. In addition, to analyze the moderated mediation effect, prior to the analysis, conscientiousness was mean-centered, the confidence level of the output confidence interval was 95%, and the number of bootstrap samples for the percentile bootstrap confidence interval was 5,000.

## 4 Results

### 4.1 Correlation between main variables

The correlations within the main constructs are shown in Table 1 through Pearson correlation analysis. Specifically, conscientiousness was positively correlated with ASE (r = 0.618, *p* < 0.001), AM (r = 0.664, *p* < 0.001), and AP (r = 0.598, *p* < 0.001). ASE was positively correlated with AM (r = 0.624, *p* < 0.001) and AP (r = 0.615, *p* < 0.001). AM was positively correlated with AP (r = 0.586, *p* < 0.001). In the meantime, the correlation coefficients between the constructs were all less than 0.7, suggesting no multicollinearity problem. In addition, regression analysis was conducted to calculate the variance inflation factor (VIF) and tolerance values, with AP as the dependent variable and conscientiousness, gender, ASE, and AM as independent variables. The results showed that the tolerance values of all independent variables were between 0.48 and 1, and the VIF value of the model was 2.079, so there was no problem of multicollinearity in the results. Furthermore, through frequency analysis, the value of conscientiousness averaged at 4.63 (range 1 to 6), ASE averaged at 3.66 (range 1 to 5), AM averaged at 5.77 (range −18 to 18), and AP averaged at 3.84 (range 1 to 5).

**Table 1:** Results of correlation and descriptive statistics analysis.

Variable	1	2	3	4
1. Conscientiousness	1			
2. ASE	0.618^***^	1		
3. AM	0.664^***^	0.624^***^	1	
4. AP	0.598^***^	0.615^***^	0.586^***^	1
M	4.6336	3.6592	5.7728	3.8413
SD	0.68351	0.6697	4.51458	0.63462

^***^p < 0.001; ASE, Academic Self-efficacy; AM, Academic Motivation; AP, Academic Performance.

### 4.2 Moderated mediation effect

**Figure 2:**
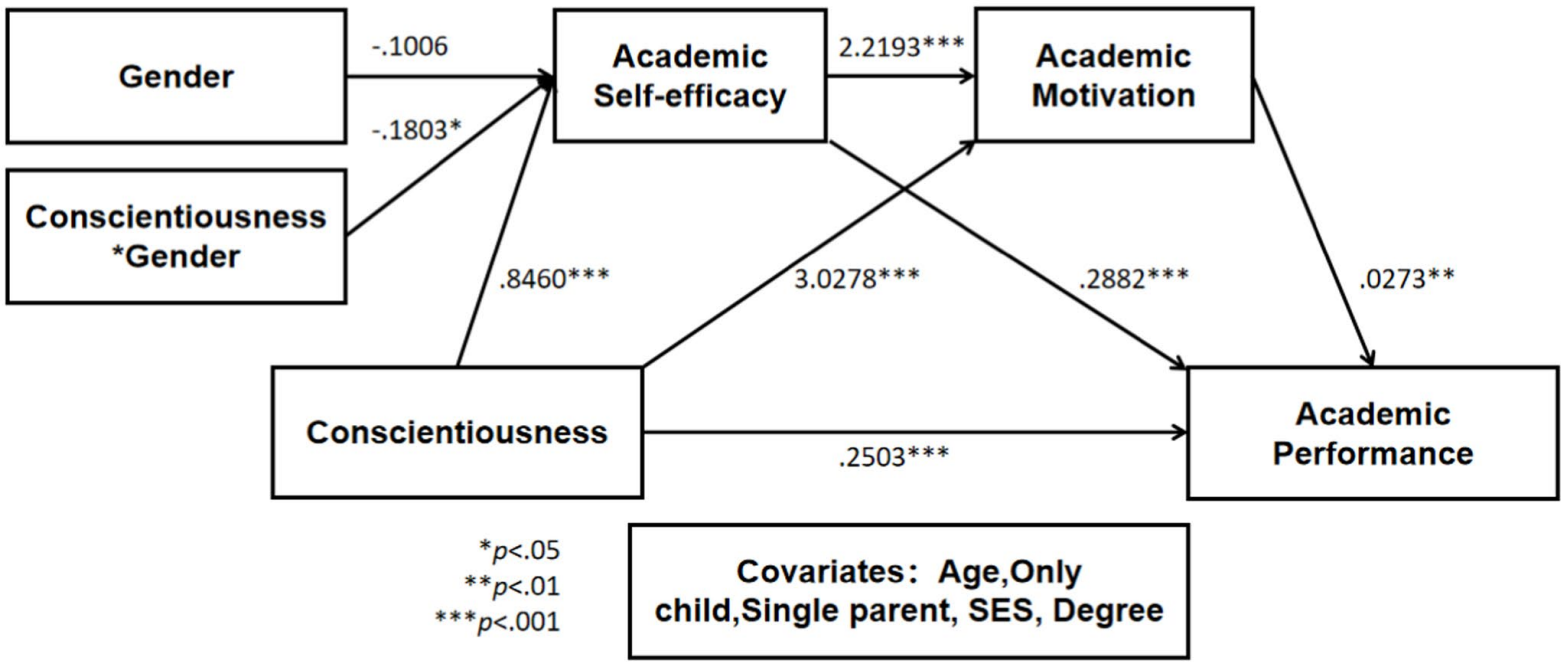
Statistical model of moderated mediation effect.

**Figure 3:**
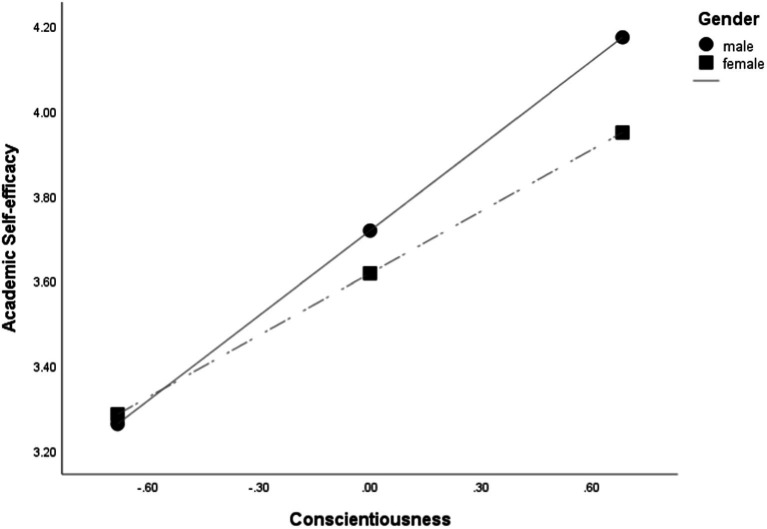
Moderating effect of gender on the relationship between conscientiousness and ASE under model 83.

To find out the moderated mediation effect of gender on the path from conscientiousness to ASE, AM, and AP, model No.83 of SPSS PROCESS macro was applied for analysis. The results are displayed in Tables 2, 3 and Figures 2, 3.

**Table 2:** Results of moderated mediation effect analysis.

Variables		Mediating variable model 1 (DV: ASE)			Mediating variable model 2 (DV: AM)			Dependent variable model (DV: AP)		
		Coeffect	SE	*t*-value	Coeffect	SE	*t*-value	Coeffect	SE	*t*-value
Constant		4.2815	0.3000	14.2711^***^	−1.7672	2.3150	−0.7634	2.7285	0.3367	8.1044^***^
ID	Conscientiousness	0.8460	0.1514	5.5886^***^	3.0278	0.3460	8.7511^***^	0.2503	0.0564	4.4388^***^
M1	ASE	–			2.2193	0.3543	6.2630^***^	0.2882	0.0548	5.2597^***^
M2	AM				–			0.0273	0.0084	3.2282^**^
W	Gender	−0.1006	0.0622	−1.6169				–		
Int	Conscientiousness*gender	−0.1803	0.0887	−2.0317^*^						
Highest order test	R^2^ change	0.0080								
	F	4.1277^*^								
Covariates	Age	0.0301	0.0267	1.1272	0.0016	0.1648	0.0096	0.0138	0.0239	0.5758
	Only child	0.0038	0.0637	0.0593	0.0097	0.3880	0.0250	0.0410	0.0564	0.7276
	Single parent	−0.0848	0.1120	−0.7574	−0.4356	0.6896	−0.6316	−0.0773	0.1003	−0.7714
	SES	−0.1439	0.0409	−3.5226^***^	−0.2743	0.2542	−1.0793	−0.0745	0.0370	−2.0146^*^
	Degree	0.0258	0.0610	0.4236	0.7016	0.3738	1.8773	0.1119	0.0546	2.0483^*^
Model summary	R^2^	0.4296			0.5228			0.4920		
	F	27.7688^***^			46.3327^***^			35.7154^***^		

Conditional effects of gender					
Gender	Effect	BOOT SE	T value	^*^LLCI	^**^ULCI
Male	0.6657	0.0722	9.2145^***^	0.5235	0.8079
Female	0.4854	0.0572	8.4850^***^	0.3728	0.5980

^*^p < 0.05; ^**^p < 0.01; ^***^p < 0.001; ID, Independent variable; DV, Dependent variable; M, Mediating variable; W, Moderating variable; ASE, Academic Self-efficacy; AM, Academic Motivation; AP, Academic Performance; Int, Interaction terms of variables. ^*^LLCI = 95% lower level of confidence intervals; ^**^ULCI = 95% upper level of confidence intervals.

**Table 3:** Analysis of direct and indirect effects.

Direct effect (conscientiousness-academic performance)				
Effect	BOOT SE	T value	^*^LLCI	^**^ULCI
0.2503	0.0564	4.4388^***^	0.1393	0.3613

Total indirect effect				
Conditional indirect effects (conscientiousness-academic self-efficacy-academic performance)				
Gender	Effect	BOOT SE	^*^LLCI	^**^ULCI
Male	0.1918	0.0447	0.1069	0.2809
Female	0.1399	0.0328	0.0786	0.2060

Index of moderation mediation				
	Index	BOOT SE	^*^LLCI	^**^ULCI
Gender	−0.0520	0.0275	−0.1102	−0.0018

Indirect effect (conscientiousness-academic motivation-academic performance)			
Effect	BOOT SE	^*^LLCI	^**^ULCI
0.0825	0.0329	0.0215	0.1527

Moderation mediating effect (conscientiousness-academic self-efficacy-academic motivation-academic performance)				
Gender	Effect	BOOT SE	^*^LLCI	^**^ULCI
Male	0.0403	0.0164	0.0105	0.0745
Female	0.0294	0.0124	0.0075	0.0557

Index of moderation mediation				
	Index	BOOT SE	^*^LLCI	^**^ULCI
Gender	−0.0109	0.0068	−0.0261	−0.0001

^***^p < 0.001.

^*^LLCI = 95% lower level of confidence intervals.

^**^ULCI = 95% upper level of confidence intervals.

First, as shown in Table 2 and Figure 2, conscientiousness positively affected ASE (0.8460, *p* < 0.001), AM (3.0278, *p* < 0.001), and AP (0.2503, *p* < 0.001). Moreover, ASE positively affected AM (2.2193, *p* < 0.001) and AP (0.2882, *p* < 0.001). Finally, AM significantly influenced AP in a positive way (0.0273, *p* < 0.01).

Second, as shown in Table 2, the interaction of conscientiousness and gender significantly affected ASE (−0.1803, *p* < 0.05), and the increase in R^2^ according to the interaction term between conscientiousness and ASE (∆R^2^ = 0.0080, *p* < 0.001) was also significant and consequently had a moderating effect. More specifically, according to the value of gender, two conditions (male and female) were given, and the conditional effect of conscientiousness on ASE according to the value of gender was found to be significant for both genders (*p* < 0.001).

The moderating effect of gender between conscientiousness and ASE was also shown in Figure 3. To confirm the pattern of interaction, the change was analyzed by dividing gender into two conditions: male and female. The degree of ASE was moderated in that conscientiousness would increase under these two conditions. When the gender was female, the slope of increasing ASE as conscientiousness increased was relatively gentle, but when the gender was male, the slope of increasing ASE as conscientiousness increased was relatively steep.

Finally, as shown in Table 3, to verify the moderated mediation effect, the conditional and non-conditional indirect effect of gender was analyzed. The direct effect of conscientiousness on AP was of significant positive effect (B = 0.2503, *p* < 0.001), and it was significant as zero did not exist in 95% of the confidence interval (0.1393 to 0.3613). Furthermore, the conditional indirect effect of ASE in the relationship between conscientiousness and AP was significant in both conditions of gender. Moreover, in the effect of conscientiousness on AP, the indirect effect of AM was of a significant positive effect (B = 0.0825), and it was significant because zero did not exist in 95% of the confidence interval (0.0215 to 0.1527). Furthermore, in the effect of conscientiousness on AP, the conditional indirect effect of ASE and AM was significant in both conditions (male and female) of gender. The adjusted index of moderated mediation was −0.0109, which was significant because there was no zero in the 95% confidence interval (−0.0261 to −0.0001). Given these results, the moderated mediating effect of male or female status was verified in the path from conscientiousness to AP, ASE, and AM.

## 5 Discussion

As the results indicated, the correlation and the moderated mediation effect among the main variables were verified. These findings are discussed further below.

First, regarding the correlation of the core variables, the results of this study were consistent with the previous studies, indicating strong positive correlations between the main variables, as expected. Specifically, in line with the previous results, the dependent variable, academic achievement, was confirmed to be positively correlated with conscientiousness (Vedel, 2014), ASE (Zeinalipour, 2022), and AM (Gnambs and Hanfstingl, 2016). Moreover, the two mediators, ASE and AM, were positively correlated with each other (McGeown et al., 2014), and they also showed positive correlations with conscientiousness (Chen et al., 2001; Apostolov and Geldenhuys, 2022). As a result, college teachers and related educators should learn and understand these personality and psychology concepts that are very relevant to AP and, further, be able to understand that these four core variables are closely related to each other. With a deeper understanding, such positive influencing factors can be applied to daily teaching activities to help students improve their performance.

Second, being consistent with the results presented in the previous study, gender moderated the path from conscientiousness to ASE, showing a major difference between male and female students (Choma et al., 2010; Miles and Naumann, 2021). Specifically, different from the previous research results of ASE focusing on arts and science (Huang, 2013), the measurement of ASE in this study was a comprehensive scale. Compared with girls, boys’ ASE grew faster as their sense of conscientiousness increased. This phenomenon suggests that for male college students, an increase in conscientiousness helps them better understand the level and capability they can achieve under their established learning goals, which exceeds that of their female classmates. This difference between male and female students provides more effective inspiration for university teachers’ teaching arrangements and students’ character development. It can guide teachers to better cultivate students’ sense of ASE.

Finally, as predicted, the mediating effects of ASE and AM were successfully validated, and the moderated mediation effect was also testified. Specifically, the mediating effect of ASE was significant for both boys and girls. In other words, ASE explained the path from conscientiousness to AP, and this effect stood for both male and female college students. This finding was in line with the previous research result (Cupani and Pautassi, 2013), suggesting socio-cognitive mechanisms such as academic self-efficacy were influential in the path from personality to academic performance. Moreover, the significant indirect effect of AM between conscientiousness and AP indicated that intrinsic and extrinsic motivation together play a role in explaining this causal relationship. These results do not rule out the possibility of other mediating variables (such as grit or growth mindset) between conscientiousness and AP, which needs to be confirmed by future research. More importantly, under the joint mediating effects of ASE and AM between conscientiousness and AP, gender still moderated such a dual mediating relationship. This illustrated that the relationship formed by conscientiousness, ASE, and AM jointly explained how they influenced students’ academic performance, and each of them played a part. In addition, gender played a significant impact on the path from the independent variable to the first mediating variable under the whole model. This research result is unique so far. It plays a pioneering role in the comprehensive use of various psychological variables in education. It also pays attention to the significant differences in gender among students. Therefore, the above results suggest that teachers can improve students’ AP while improving their sense of conscientiousness. Students’ ASE should also be improved through various means, and targeted treatment can be given to students of different genders. For example, specific course schedules or activities can be designed with gender-based grouping in advance to achieve better teaching outcomes. Particularly, male students’ academic self-efficacy will be better improved than female students with the improvement of conscientiousness in teaching activities. In addition, students’ learning motivation needs to be better guided, and they need to have stronger intrinsic motivation and cooperate with viable extrinsic motivation interventions. The implementation of the above methods under a common educational concept can improve students’ overall academic achievement.

## 6 Conclusion

In conclusion, the current study proved the moderated mediation effect of gender on the path from conscientiousness to academic performance through the dual mediation effect of academic self-efficacy and academic motivation.

As for the limitations of this study, first, the university student samples used in this article are limited to China. More research is needed on the relevant situations of college students in other countries or regions to have a more comprehensive understanding of the situation worldwide. Second, the academic motivation scale includes two main aspects, intrinsic motivation and extrinsic motivation, and seven sub-dimensions. This article failed to conduct further sub-dimension analysis, which made explaining the mediating mechanism of learning motivation less thorough.

In general, this article is the only one known to date that uses simple random sampling to verify the moderated mediating effect of gender in the relationship from conscientiousness to academic self-efficacy, academic motivation, and academic performance. The research findings of this article provided new perspectives and inspirations for the study of specific fields in this research.

For related suggestions, as the understanding of the factors contributing to academic performance and the relationship between them deepens, university teachers and organizers of university education should increase the teaching links of honing students’ character qualities in the education system, regularly introduce lectures on educational psychology-related knowledge to students, and carry out more practical activities to exercise and improve their academic self-efficacy. At the same time, they can guide students to set higher ambitions and thus enhance their intrinsic motivation. In addition, educators should also receive related training regularly to improve their relevant academic level, familiarize themselves with the latest psychological theoretical discoveries, and apply them to educational measures. In addition, governments and education policymakers in various countries should also continuously integrate these positive psychology research results into education reform measures. In this sense, the research results of this article will play a key role in future education optimization practices and enable students to be truly improved.

As for future research directions, longitudinal research should be conducted to deepen the results of this study if conditions permit. In addition, it is recommended to explore more operational moderating variables based on this model to effectively intervene in students’ academic situations.

## Data Availability

The raw data supporting the conclusions of this article will be made available by the authors, without undue reservation.
